# Neuroanatomical characterization of the Nmu-Cre knock-in mice reveals an interconnected network of unique neuropeptidergic cells

**DOI:** 10.1098/rsob.220353

**Published:** 2023-06-14

**Authors:** Mireia Medrano, Wissal Allaoui, Mathias Van Bulck, Sofie Thys, Leila Makrini-Maleville, Eve Seuntjens, Winnok H. De Vos, Emmanuel Valjent, Bálazs Gaszner, Ann Van Eeckhaut, Ilse Smolders, Dimitri De Bundel

**Affiliations:** ^1^ Center for Neurosciences, Department of Pharmaceutical Chemistry, Drug Analysis and Drug Information, Research Group Experimental Pharmacology, Vrije Universiteit Brussel, 1090 Brussels, Belgium; ^2^ Laboratory of Medical and Molecular Oncology, Vrije Universiteit Brussel, 1090 Brussels, Belgium; ^3^ Department of Veterinary Sciences, Laboratory of Cell Biology and Histology and Antwerp Centre for Advanced Microscopy (ACAM), University of Antwerp, 2610 Antwerp, Belgium; ^4^ μNEURO Research Centre of Excellence, University of Antwerp, 2610 Antwerp, Belgium; ^5^ IGF, Université de Montpellier, CNRS, Inserm, 34094 Montpellier, France; ^6^ Department of Biology, Laboratory of Developmental Neurobiology, KU Leuven, 3000 Leuven, Belgium; ^7^ Antwerp Centre for Advanced Microscopy (ACAM), 2610 Wilrijk, Belgium; ^8^ Medical School, Research Group for Mood Disorders, Department of Anatomy and Centre for Neuroscience, University of Pécs, 7624 Pécs, Hungary

**Keywords:** neuromedin U, neuropeptide, knock-in mouse model, ventromedial hypothalamic nucleus

## Abstract

Neuromedin U (NMU) is an evolutionary conserved neuropeptide that has been implicated in multiple processes, such as circadian regulation, energy homeostasis, reward processing and stress coping. Although the central expression of NMU has been addressed previously, the lack of specific and sensitive tools has prevented a comprehensive characterization of NMU-expressing neurons in the brain. We have generated a knock-in mouse model constitutively expressing Cre recombinase under the *Nmu* promoter. We have validated the model using a multi-level approach based on quantitative reverse-transcription polymerase chain reactions, *in situ* hybridization, a reporter mouse line and an adenoviral vector driving Cre-dependent expression of a fluorescent protein. Using the Nmu-Cre mouse, we performed a complete characterization of NMU expression in adult mouse brain, unveiling a potential midline NMU modulatory circuit with the ventromedial hypothalamic nucleus (VMH) as a key node. Moreover, immunohistochemical analysis suggested that NMU neurons in the VMH mainly constitute a unique population of hypothalamic cells. Taken together, our results suggest that Cre expression in the Nmu-Cre mouse model largely reflects NMU expression in the adult mouse brain, without altering endogenous NMU expression. Thus, the Nmu-Cre mouse model is a powerful and sensitive tool to explore the role of NMU neurons in mice.

## Graphical abstract

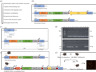

## 1. Introduction

Neuromedin U (NMU) is recognized as a multifunctional neuropeptide involved in various physiological processes, such as circadian regulation, energy homeostasis and regulation of feeding behaviour, reproductive behaviours, reward processing and stress coping [1–4]. However, knowledge on the distribution, expression and connectivity of NMU-producing cells in the brain remains limited. NMU is coded by the *Nmu* gene and shows a notable amino acid sequence homology across different species [3]. The high degree of amino acid sequence conservation is indicative of a strong evolutionary pressure to maintain its structure and function across not only mammals but the entire vertebrate subphylum [1–5]. Moreover, NMU analogues are conserved across bilaterian animals, including invertebrates [6–8]. In mammals, NMU exists mainly in two principal forms, a long amino acid peptide (NMU-23 in rodents, NMU-25 in humans) and a truncated peptide (NMU-8), although other forms have also been reported [2,9]. The asparagine-linked amidation at the C-terminus of NMU is an important structural characteristic of the peptide. Indeed, this post-translational modification has been shown to be indispensable for receptor binding and activation [10–13]. NMU exerts its biological effects via two known G protein-coupled receptors: NMUR1, which is mainly expressed in the periphery; and NMUR2, principally expressed in the central nervous system (CNS) [1].

NMU distribution in the CNS has been investigated in different species including human, rat and to a lesser extent in mouse. These studies used chromatographic techniques, radioimmunoassays (RIAs) and immunohistochemical (IHC) analyses to detect NMU-like immunoreactivity (NMU-LI) [14–22], and *in situ* hybridization (ISH), reverse-transcription quantitative polymerase chain reaction (RT-qPCR) and Northern blot analysis to study *Nmu* mRNA distribution [10,11,15,23–32]. Compared to expression levels in the small intestine, low or moderate levels of NMU-LI have been reported in the CNS, both in the brain and in the spinal cord [1,3]. Within the brain, the highest levels of NMU-LI were detected in rat, human and mouse pituitary gland [16,19,22]. Moderate to high levels of *Nmu* mRNA and NMU-LI were found in the hypothalamus [16,19,21,25,26,32], with highest levels in the mouse suprachiasmatic nucleus (SCh), dorsomedial hypothalamic nucleus (DMH), arcuate nucleus (ARC) and ventromedial hypothalamic nucleus (VMH) [26], supporting the described role of NMU in circadian regulation, reproduction, and feeding and energy homeostasis [1–4]. However, it should be noted that previous descriptions of NMU expression in the CNS show a certain level of inconsistency. Histochemical techniques often have insufficient sensitivity to detect low-abundant molecules and the currently commercially available anti-NMU antibodies show low specificity. Furthermore, most IHC studies used primary antisera raised against synthetic porcine NMU-8, using colchicine to block axonal transport and increase the peptide content in cell bodies [16,20,22]. Potential cross-reactivity and inherent differences between antiserum batches could partially explain the lack of reproducible results regarding NMU distribution. Moreover, a previous RIA analysis showed interspecies molecular differences of NMU-LI [18], pointing out that the NMU distribution reported in different species using only synthetic porcine NMU-8 should be analysed with caution. In addition, colchicine pretreatment can alter normal brain physiology, limiting its use for studying peptide distribution under normal conditions [33]. Moreover, classical ISH techniques lack the sensitivity required to detect many low-abundant transcripts [34,35]. Indeed, a few studies have addressed *Nmu* mRNA expression by ISH in mice, showing relevant discrepancies in terms of level of expression and distribution [10,21,26,36]. Therefore, a more detailed and unambiguous description of the distribution, expression and connectivity of NMU-producing cells in the brain would enhance our understanding on the central role of NMU in health and disease.

Over the past two decades, technical developments have created opportunities to access neural systems at a molecular, cellular, circuit and functional level [37,38]. Bacterial artificial chromosome (BAC)-mediated transgenics and gene targeting are approaches that are widely used to drive the expression of specific genes of interest [38,39]. One of the most extensively used cell-type-specific genetic targeting strategies is the binary system Cre/LoxP. In this system, the bacteriophage recombinase Cre transgene, of which the expression is under the regional control of specific gene promotors, controls the expression of a target gene by inducing the recombination between two LoxP sites [38,40,41]. Mice that express Cre under a cell-type-specific promoter are instrumental for functional neuroanatomical characterization. Cre drivers can be cross-bred with reporter lines that carry a fluorescent protein to provide cell-specific expression of the fluorescent marker. Moreover, the Cre driver can be targeted with Cre-dependent viral vectors to manipulate cells of interest in a temporal and regional-specific manner. This permits the elucidation of the neuroanatomical organization and functions of the targeted cells. At present, two BAC transgenic mice have been generated driving the Cre recombinase gene or the enhanced green fluorescent protein (EGFP) reporter gene under the *Nmu* promoter but they have not yet been systematically validated [42]. In addition, it should be noted that BAC transgenes are generated by non-specific integration of the transgene into the target genome. Therefore, the transgene can be inserted into an unknown locus in the genome, leading to ectopic expression and affecting the expression of neighbouring genes [43]. Moreover, expression variegation makes it necessary to functionally screen founder lines [43,44], since the positional effect may cause a variable number of copies to be incorporated into the genome, thus affecting the level of Cre expression [38,43–45].

In the present study, we generated and validated a novel Nmu-Cre knock-in mouse model constitutively expressing Cre recombinase under the endogenous *Nmu* promoter. The validation was ensured by cross-breeding the Nmu-Cre mouse with a reporter line and by detecting *Nmu* mRNA expression using RT-qPCR and ISH. We performed a detailed neuroanatomical characterization of NMU-expressing cells in the mouse brain and characterized these cells using a viral tracer, IHC and ISH analysis.

## 2. Material and methods

### 2.1. Animals

The B6.NmuCre-IRES-Nmu or Nmu-Cre knock-in mouse line was developed in collaboration with genOway (France) on a C57BL/6 genetic background as described below. PCR-selected male and female Nmu-Cre heterozygous mice were backcrossed to C57BL/6J mice (Janvier, France) to maintain the breeding colony. B6.Cg-*Gt(ROSA)26Sor^tm6(CAG–ZsGreen1)Hze^*/J, also known as Ai6 or Ai6(RCL-ZsGreen) [41] mice (stock no. 007906, The Jackson Laboratory, USA; RRID IMSR_JAX:007906) were used as Cre reporter line. This line contains a loxP-flanked STOP cassette preventing transcription of a CAG promoter-driven EGFP variant (ZsGreen1). PCR-selected male and female Nmu-Cre heterozygous mice were crossed with homozygous Ai6 reporter mice to obtain Nmu-Cre:ZsGreen1 offspring that drive the expression of the green fluorescent protein ZsGreen1 in a Cre-dependent manner. All mice were bred in-house at the animal facility of the Vrije Universiteit Brussel. All mice were adult (greater than or equal to eight weeks) when set in breeding or at the start of experiments. Mice were group-housed (1290 Eurostandard type III cages, Tecniplast, Italy) in a temperature (18–24°C) and humidity (30–70%) regulated environment with a 12L : 12D h cycle (onset dark cycle: 18.00). Mice had free access to food pellets (A03, SAFE, France) and water. Cages were minimally enriched with shelters, wooden gnawing blocks and nesting material. Nmu-Cre mice that underwent stereotaxic surgery were singly housed afterwards (1264C Eurostandard type II cages, Tecniplast, Italy) for the remainder of the experiment. All mice used in the present study were sacrificed between 15.00 and 17.00 (light cycle). All experiments were executed by certified and experienced researchers and were approved by the Ethical Committee for Animal Experiments of the Faculty of Medicine and Pharmacy of the Vrije Universiteit Brussel. All experiments were performed according to the European Community Council directive (2010/63/EU) and the Belgium Royal Decree (29 May 2013), and complied with the ARRIVE guidelines [46]. All efforts were made to reduce stress and suffering of the animals to a minimum.

### 2.2. Generation of the Nmu-Cre knock-in mouse model

**Figure 1.**
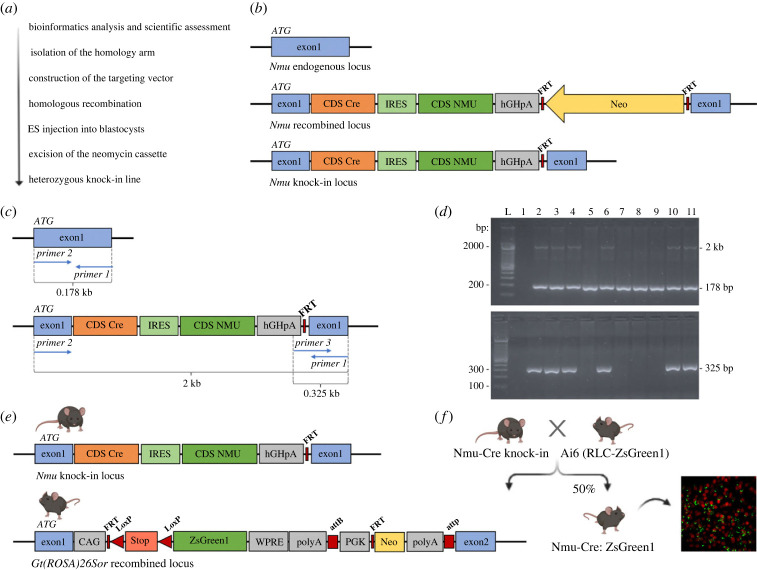
The generation of the Nmu-Cre knock-in mouse model. (*a*) Step by step progression for the generation of the knock-in line. (*b*) Schematic representation of the selected *Nmu* gene targeting strategy. The Cre-IRES-*nmu*-hGHpolyA cassette was inserted in frame with exon 1 ATG of the *Nmu* gene. The neomycin selection cassette was excised to generate heterozygous mice carrying the neo-excised *nmu* knock-in allele. (*c*) Diagram representing *Nmu* endogenous locus (top) and knock-in locus (bottom) with the binding sites of the screening primers. (*d*) Representative PCR results from genotyped animals. After gel electrophoresis, a distinction could be made between wild-type and heterozygous genotypes. PCR products of 178 bp, 325 bp and 2 kb (lines 2–4, 6, 10 and 11) corresponding to the heterozygous genotype; PCR products of 178 bp (lines 5, 7–9) corresponding to the wild-type genotype. L: ladder; line 1: negative control. (*e*) Schematic representation of the *Nmu* knock-in locus in Nmu-Cre mice (top) and the *Gt(ROSA)26Sor* recombined locus in Ai6 reporter mice (bottom). (*f*) Representation of the cross between Nmu-Cre knock-in mice and Ai6 reporter mice. The resulting offspring, Nmu-Cre:ZsGreen1, expresses the green fluorescent protein ZsGreen1 in all cells expressing Cre recombinase. Diagrams are not depicted to scale. IRES: internal ribosome entry site; CDS: coding sequence; FRT: flippase recognition target sites; Neo: neomycin resistance gene; WPRE: woodchuck hepatitis virus post-transcriptional regulatory element; PGK: phosphoglycerate kinase selection marker; attB/attP: specific recombination sites; LoxP: locus of X(cross)-over in P1.

The selected targeting strategy for generation of the Nmu-Cre knock-in mouse model (figure 1) consisted of the insertion of a Cre-internal ribosome entry site (IRES)-*Nmu* complementary DNA (cDNA) in frame with exon 1 ATG of the *Nmu* gene on chromosome 5qC3.3, maintaining the expression of the most prevalent isoform encoding for the 174 amino acid precursor and minimizing both the potential risk of interfering with regulatory elements and the deregulation of the targeted and/or neighbouring genes. The Cre-IRES-*Nmu* targeting vector was designed (from 5′ to 3′) with a Cre coding sequence, an IRES element, the coding sequence of NMU isoform 1, an hGHpolyA signal and a neomycin cassette for positive selection, flanked by two flippase recognition target (FRT) sites (figure 1*b*). To generate the targeting vector carrying the Cre cassette, two homology arms were first isolated from the *Nmu* gene of C57BL/6J mouse genomic DNA and were validated by sequencing. A positive control vector for subsequent PCR screening assays was also generated, in this case mimicking the DNA configuration of the targeted locus after the homologous recombination event between the short homology arm and the *Nmu* locus. The linearized targeting vector was electroporated into embryonic stem (ES) cells, which were then subjected to positive selection. The ES cell line used is a proprietary C57BL/6N ES cell line routinely used by genOway for mouse model production. The resistant ES cell clones were first subjected to a PCR screening over the 3′ short homology arm, using a primer hybridizing within the neomycin cassette and a primer hybridizing downstream of the 3′ homology arm. The optimized PCR protocol was tested in wild-type DNA from C57BL/6J mice as a negative control, and in the positive control vector in the presence of wild-type C57BL/6J genomic DNA. The clones validated by PCR were further analysed by Southern blot to validate the correct recombination event at the 5′ end of the targeted locus, using a neomycin (internal) probe. No additional randomly integrated copy of the targeting construct was reported in the selected ES clones. The correct 3′ recombination event was also further assessed by Southern blot, in this case using an external probe. The detection of two restriction fragments indicated the heterozygous status of the recombined *Nmu* locus. The PCR- and Southern blot-positive recombined ES clones, further validated by sequencing analysis to ascertain the integrity of the inserted cassettes, were injected into blastocysts to obtain chimeric mice carrying the recombined locus. Highly chimeric males assessed by coat colour marker comparison were placed in breeding to ensure germline transmission. The selected chimeric animals were crossed with C57BL/6J Flp deleter mice, in order to excise the neomycin selection cassette and generate heterozygous mice carrying the neo-excised knock-in allele. The Flp line was developed at genOway by pronuclear injection of a CMV-Flp encoding sequence. The Flp deleter line displays ubiquitous the expression of Flp recombinase and efficient FRT-flanked sequence recombination. It has been used for several years for neomycin cassette removal. The excision of the neomycin selection cassette prevents unwanted interactions between the cassette and control regions of the targeted locus, thus disrupting the expression of neighbouring genes within the locus [47]. The Flp transgene was bred out from the mouse line, as confirmed by PCR following the removal of the neomycin cassette. Progeny was then genotyped by PCR and the excision event, and the integrity of the targeted region was further verified by sequence analysis on a subset of the PCR-validated animals.

### 2.3. Genotyping

Mutant mice were offspring of the cross between Nmu-Cre knock-in and C57BL/6J mice or between Nmu-Cre knock-in and Ai6 reporter mice. Genotypes of the offspring were verified by PCR amplification of genomic ear DNA using the commercially available REDExtract-N-Amp™ Tissue PCR Kit (no. R4775; Sigma-Aldrich, Germany) and primers designed to identify the presence or absence of the knock-in allele. The following primer couples were used: 5′-GTGACAGGAGAGGAGATGCGGTTGC-3′ (forward primer) and 5′-AGCAAGAGGAGGCGCACAGGA-3′ (reverse primer) to detect the wild-type allele (178 bp); and 5′-GTGACAGGAGAGGAGATGCGGTTGC-3′ (forward primer) and 5′-ACCTTGGCCTCCCAAATTGCTG-3′ (reverse primer) to detect the neo-excised knock-in allele (325 bp) (figure 1*c*,*d*). All primers were obtained from Eurogentec (Belgium). The PCR amplification reaction took place at the following conditions: 94°C for 2 min, 30 cycles of 94°C for 30 s, 65°C for 30 s and 68°C for 5 min, and a final extension step at 68°C for 8 min. PCR reaction products were separated on a 2% agarose electrophoresis gel and visualized with GelRed (nucleic acid gel stain; no. 41003, Biotium, USA).

### 2.4. Viral transduction

Viral transduction of NMU-expressing cells was achieved by intracerebral injection of the ready-to-use double-floxed adeno-associated viral (AAV) vector AAV5-EF1a-DIO-RFP (Vector Biosystem, USA), driving Cre-dependent expression of the red fluorescent protein (RFP) in a temporally and regionally defined manner. Hereto, mice were deeply anaesthetized in an induction chamber with 2–3% isoflurane (1000 mg g^−1^, Vetflurane Neurology, Virbac, Belgium) and mounted on a stereotaxic frame. Anaesthesia was maintained during the entire duration of the surgery using 1–2% isoflurane in 100% oxygen, delivered via an inhalation cone. Meloxicam (5 mg kg^−1^, Metacam^®^, 5 mg ml^−1^, Boehringer Ingelheim, Germany) was administered subcutaneously to prevent post-operative pain and inflammation. Artificial tear ointment (Duratears^®^, Novartis, Belgium) was applied on the eyes to prevent dehydration. After disinfection of the skin with iso-Betadine^®^ (Mylan, Belgium), an incision was made, the skull was cleared and small holes were drilled (Volvere Vmax, NSK, Germany) at specific coordinates relative to Bregma (VMH: antero-posterior (AP) –1.94 mm, medial-lateral (ML) ± 0.4 mm and dorsoventral (DV) −5.75 mm; V1: AP −2.92 mm, ML ± 2.3 mm, DV −0.6 mm). Next, the AAV vector was bilaterally infused into the target regions (titre 1.2 × 10^12^ gc ml^−1^; 500 nl into the VMH, 400 nl into the V1) at a flow rate of 0.15 µl min^−1^ using a 10 µl microsyringe (Hamilton Neuros, USA) kept vertical to the plane of the brain in a microinjector unit (Model 5001, Kopf^®^, USA). The syringe was kept in place for an additional 10 min to limit backflow along the injection track. After injections, the skin was closed using non-absorbable suture (Ethilon II, 4-0, M-2, Ethicon, USA). At the end of the surgical procedure, mice received 1 ml saline (0.9% NaCl, Baxter, Belgium) intraperitoneally to prevent dehydration and were placed in a recovery box with heating (ThermaCage^®^, Datesand Group, UK) until fully awake and responsive. After surgery, mice were returned to their home cage to allow further recovery. Mice were singly housed to prevent damage to the suture and sacrificed for *ex vivo* experiments three weeks after surgery to ensure proper transduction of the AAV vector and full expression of RFP.

### 2.5. Tissue preparation for histology

Mice were deeply anaesthetized by an intraperitoneal overdose of sodium pentobarbital (250 mg kg^−1^ Dolethal^®^, Vetoquinol, France). Immediately after respiratory arrest, transcardial perfusion was performed using phosphate-buffered saline (PBS, pH 7.4, Sigma-Aldrich, Germany) followed by 4% paraformaldehyde (PFA, VWR International, Belgium) in PBS (pH 7.4) for 5 min at a rate of 10 ml min^−1^. After transcardial perfusion, brains were dissected and post-fixated overnight with 4% PFA and then stored in Tris-buffered saline (TBS) solution (50 mM Tris, pH 7.6, Sigma-Aldrich, Germany) at 4°C. Forty micrometre coronal sections were sliced using a vibratome (Leica VT1000S, Leica Biosystems, Germany) and stored at −20°C in an anti-freeze solution (30% glycerol (Merck Millipore, Germany), 30% ethylene glycol (VWR International, USA) and 10% TBS) until further processing. Free-floating sections of specific brain areas were selected and rinsed three times with TBS, each for 10 min. Next, sections were pretreated with TBS containing 0.1% Triton-X (TBS-T; Sigma-Aldrich, Germany) for 15 min at room temperature and were then incubated with 25 µg ml^−1^ of 4′,6-diamidino-2-phenylindole dihydrochloride (DAPI; Cell Signaling Technology, USA) for 5 min. Finally, the slices were washed two times with Tris-buffer (TB, 50 mM Tris, pH 7.6, Sigma-Aldrich, Germany), mounted on Superfrost slides (Superfrost plus, VWR International, Belgium) and coverslipped using Dako mounting medium (Agilent, USA). Endogenous ZsGreen1 fluorescence and DAPI staining were evaluated using a fluorescence microscope (Evos FL Auto, Thermo Fisher Scientific, USA) and analysed using Image J (NIH, USA; RRID:SCR_003070).

### 2.6. Immunohistochemistry

Free-floating 40 µm coronal sections were selected and rinsed three times for 10 min with TBS. Next, sections were pretreated with TBS-T containing 10% donkey serum (Merck Millipore, USA) for 1 h at room temperature under gentle agitation. Next, sections were incubated overnight in primary antibodies (table 1) at 4°C. The next day, sections were rinsed three times with TBS-T and incubated with secondary antibodies (table 2) for 45 min at room temperature and protected from light. DAPI staining and mounting were performed as described in §2.5. Fluorescent labelling was visualized with a fluorescence microscope (Evos FL Auto, Thermo Fisher Scientific, USA) and a confocal laser scanning microscope (Zeiss, Axio Observer with LSM 710-6NLO configuration, Zeiss International, Germany), and analysed using Image J (NIH, USA; RRID:SCR_003070). DAPI staining was used to identify fibre tracts, ventricles and reference regions and estimate the antero-posterior levels relative to Bregma in each slice. Large scans of coronal brain slices and higher magnification images were aligned with the boundaries defined in the brain atlas of Paxinos & Franklin [48] using the *BigWarp* plugin from ImageJ to delineate regions and estimate the extent of the fluorescence signal (electronic supplementary material, figure S3). The specificity of the anti-proopiomelanocortin (POMC) antibody was verified earlier [49]. The specificity of all the other primary antibodies used in this study was determined by Western blot analysis (see suppliers' information; table 1).

**Table 1.** Primary antibodies.

antibody	class	host species	manufacturer (catalogue no.)	dilution	RRID^a^
anti-glial fibrillary acidic protein (GFAP)	polyclonal	chicken	OriGene, USA (TA309150)	1 : 1000	
anti-oligodendrocyte transcription factor 2 (Olig2)	polyclonal	rabbit	Merck Millipore, USA (AB9610)	1 : 1000	AB_570666
anti-neuronal nuclear protein (NeuN)	polyclonal	guinea-pig	Synaptic Systems, Germany (266004)	1 : 400	AB_2619988
anti-ionized calcium-binding adaptor molecule 1 (Iba1)	monoclonal	rabbit	Thermo Fisher Scientific, USA (MA5-27726)	1 : 1000	AB_2735228
anti-mCherry	polyclonal	goat	LifeSpan BioSciences, USA (LS-C204207)	1 : 500	AB_2619713
anti-estrogen receptor alpha (ER*α*)	polyclonal	rabbit	Merck Millipore, USA (06-935)	1 : 1000	AB_310305
anti-proopiomelanocortin (POMC)	polyclonal	rabbit	Phoenix Pharmaceuticals, USA (H029-30)	1 : 400	AB_2307442
anti-neuropeptide Y (NPY)	monoclonal	mouse	Santa Cruz, USA (sc-133080)	1 : 500	AB_2298657

^a^RRID: Research Resource Identifier; https://scicrunch.org/resources.

**Table 2.** Secondary antibodies. All secondary antibodies used were ordered from Jackson ImmunoResearch (USA).

conjugate	target	host species	catalogue no.	dilution	RRID^a^
*Cyanine Cy™3*	chicken	goat	103-165-155	1 : 500	AB_2337386
*Cyanine Cyy™5*	rabbit	donkey	711-175-152	1 : 400	AB_2340607
*Cyanine Cyy™5*	guinea-pig	donkey	706-175-148	1 : 500	AB_2340462
*Cyanine Cyy™3*	rabbit	donkey	711-165-152	1 : 400	AB_2307443
*Cyanine Cyy™3*	goat	donkey	705-165-147	1 : 400	AB_2307351
*Cyanine Cyy™5*	mouse	goat	115-175-146	1 : 400	AB_2338713
				1 : 800	

^a^RRID: Research Resource Identifier; https://scicrunch.org/resources.

### 2.7. Preparation of optically cleared brains

#### 2.7.1. Sample preparation and delipidation

Mice were perfused and their brains were collected and post-fixed as described above (§2.5). Post-fixed brains were rinsed with PBS at least two times for 2 h at room temperature. Brains were then delipidated with clear unobstructed brain imaging cocktail and computational analysis (CUBIC)-L clearing solution (10 wt% *N*-butyldiethanolamine (no. 471240, Sigma-Aldrich, Germany) and 10 wt% Triton X-100 (Sigma-Aldrich, Germany) in Milli-Q water) [50,51]. To do so, brains were first immersed in 1 : 1 Milli-Q water-diluted CUBIC-L with gentle shaking (75 rpm) at 37°C overnight. Next, the samples were incubated in complete CUBIC-L for 10–12 days, with gentle shaking (75 rpm) at 37°C. CUBIC-L was refreshed every 2–3 days. Brains were then washed three times for 2 h with PBS at room temperature.

#### 2.7.2. Refractive index matching and agarose gel embedding

Refractive index matching was achieved by incubating the cleared brains in CUBIC-R + (M) (45 wt% 2,3-dimethyl-1-phenyl-5-pyrazole (antipyrine; no. 10784, Sigma-Aldrich, Germany), 30 wt% *N*-methylnicotinamide (no. M0374, TCI, Japan) and 0.5 wt% *N*-butyldiethanolamine (no. 471240, Sigma-Aldrich, Germany), pH 9.6–9.8 in Milli-Q water) [51,52]. To do so, the samples were first immersed in 1 : 1 Milli-Q water-diluted CUBIC-R + (M) with gentle shaking (75 rpm) at room temperature overnight. The next day, the solution was replaced by complete CUBIC-R + (M) and samples were further incubated for 24 h. Gel embedding was based on Matsumoto and colleagues [53]. Briefly, agarose (2% w/v) was thoroughly diffused in fresh CUBIC-R + (M) (adjusted pH 8.5–9) and the mixture was then incubated in a hot water bath at 60°C during the whole embedding protocol. A bottom gel layer was formed in a custom-made mould and was incubated at room temperature for 15 min. Then, the mixture was poured into a 15 ml falcon tube, the sample was added and immediately decanted into the gel mould, positioning the sample in the centre and the desired orientation. Bubbles, if present, were carefully removed with a pipette tip and the gel was incubated at room temperature for another 15 min. To prepare the top layer, agarose–CUBIC-R + (M) solution was decanted into the gel mould until the surface protruded slightly. Bubbles were carefully removed, and the embedded sample was incubated at room temperature for at least 4 h.

#### 2.7.3. Refractive index matching with ethyl cinnamate

The samples were gently removed from the mould and incubated for 30 min in fresh CUBIC-R + (M) (adjusted pH 8.5–9). Then, the brains were dehydrated in increasing concentrations of 1-propanol pH 9 (1-propanol in CUBIC-R+; 25, 50, 75, 2 × 100%, 30 min each step and a final overnight step at 100%). The next day, dehydrated samples were incubated in increasing concentrations of ethyl cinnamate (ECi, no. 112372, Sigma-Aldrich, Germany; ECi in 1-propanol pH 9; 25, 50, 75, 2 × 100%, 45 min each step) [54–56] and were kept in 100% ECi until image acquisition in a light-sheet fluorescence microscope (LSFM).

#### 2.7.4. Light-sheet fluorescence microscope image acquisition and processing

Image acquisition was done in ECi using a Lavision Ultramicroscope II (Lavision Biotec, Germany) equipped with an Olympus MVPLAPO 2× (NA 0.50) objective lens and DBE-corrected LV OM DCC20 dipping cap. Images were recorded with a Neo sCMOS camera (Andor), using a 2× objective and a digital zoom of 0.63×. Samples were optically sectioned using a z-step size of 10 µm, resulting in 5.08 µm × 5.08 µm × 10 µm voxel size (16 bit per pixel). A 488 nm laser and 561 nm laser were used in combination with a 525/50 nm and 620/60 nm emission filter, respectively. Sagittal optical sections were acquired in a mosaic of four tiles using the left and right light sheets separately, respectively, for the left and right parts of the mosaic. The resulting image sets were then imported into Arivis Vision 4D software (Zeiss, Germany; RRID SCR_018000) for stitching and acquisition of movies, images and three-dimensional renderings.

### 2.8. RNA *in situ* hybridization

#### 2.8.1. Chromogenic detection

Adult Nmu-Cre mice were perfused as described in §2.5, using 10% neutral-buffered formalin (NBF) as a fixative. After transcardial perfusion, brains were dissected and post-fixated for 24 h with 10% NBF at room temperature in a rotating shaker, followed by standard paraffin embedding using an automatic tissue processor (Leica TP 1020, Leica Microsystems, Germany). Brains were sectioned with a microtome at 5 µm thickness. The RNAscope^®^ 2.5 HD Assay-RED (no. 322350, Advanced Cell Diagnostics, USA) was used according to the manufacturer's instructions using adapted pretreatment conditions (15 min Target Retrieval and 5 min Protease Plus). Probes used were *Bacillus subtilis* dihydrodipicolinate reductase (dapB, no. 310043), *Mus musculus* peptidyl-prolyl isomerase B (Ppib, no. 313911) and *Mus musculus* Nmu (no. 446831). Slides were counterstained for haematoxylin and mounted with VectaMount^®^ mounting medium (Vector Laboratories, USA). Images were acquired with a brightfield microscope (Olympus BX61, Japan) at 20× and 40× magnification. Haematoxylin staining was used to identify fibre tracts and reference regions to estimate the antero-posterior levels relative to Bregma in each slice.

#### 2.8.2. Fluorescent multiplex detection

Adult Nmu-Cre mice were sacrificed by cervical dislocation, and brains were removed, placed immediately on dry ice for 5 min and stored at −80°C. Brains were sectioned at −17°C with a cryostat at 14 µm thickness. Slices were collected onto Superfrost Plus slides (Thermo Fisher Scientific, USA). Probes for *Nmu* (no. 446831-C3), *Slc32a1* (no. 319191-C2) and *Slc17a6* (no. 319179-C1) were used with the RNAscope^®^ Fluorescent Multiplex labeling kit (no. 320850; Advanced Cell Diagnostics) according to the manufacturer's recommendations. Slides were mounted with ProLong Diamond Antifade mountant (no. P36961, Invitrogen, USA). Single-molecule fluorescence of labelled cells was captured using sequential laser scanning confocal microscopy (Leica SP8, Germany) and analysed using Image J (NIH, USA; RRID:SCR_003070). DAPI staining was used to identify fibre tracts, ventricles and reference regions and estimate the antero-posterior levels relative to Bregma in each slice.

### 2.9. Quantitative reverse-transcription polymerase chain reaction

RNA extraction and RT-qPCR were performed as previously reported [57] with slight modifications. Mice were sacrificed by cervical dislocation and brains were rapidly removed from the skull. The brain was placed ventral side up on top of a Petri dish filled with ice. Using curved forceps, the hypothalamus was isolated by pinching it out while pushing down with the forceps. The hemispheres were separated and the caudate putamen, identified by its characteristic striated appearance, was gently separated, revealing the hippocampus–white matter–cortex region. The hippocampus, identified by its lighter colour and curved shaped, was carefully isolated using the curvature of the blade. The tissue was immediately snap-frozen in 2-methylbutane (J.T. Baker, Poland) and stored at −80°C. All surgical instruments were cleaned with RNAseZap (Thermo Fisher Scientific, USA) in between each animal and brain region. Extraction and purification of total RNA were performed using the RNeasy Mini Kit (no. 74104, Qiagen, Germany) according to the manufacturer's instructions. A Nanodrop spectrophotometer (Thermo Scientific, USA) was used to check relative RNA concentration and purity, measuring both 260/280 nm (approx. 2.0) and 260/230 nm (approx. 2.0–2.2) ratios. Following extraction, cDNA synthesis (iScript cDNA synthesis kit, Biorad, Belgium) and cDNA purification (Genelute PCR clean-up kit, Sigma-Aldrich) were carried out, according to the manufacturer's instructions. qPCR was performed using SYBR^TM^ Green PCR master mix (no. 4309155, Applied Biosystems^TM^, Thermo Fisher Scientific, USA). Specific primers for the gene of interest *Nmu* (NCBI reference sequence: NM_019515.1; forward 5′-ATCTGTTGCACTGAGGGAGC-3′; reverse 5′-TCATGCAGTTGAGGGACGAG-3′) and the reference genes beta-2 microglobulin (*B2m*, NCBI reference sequence: NM_009735-3; forward 5′-TTCTGGTGCTTGTCTCACTGA-3′; reverse 5′-CAGTATGTTCGGCTTCCCATTC-3′) and hypoxanthine guanine phosphorybosyl transferase 1 (*Hprt1*, NCBI reference sequence: NM_013556.3; forward 5′-CAAACTTTGCTTTCCCTGGT-3′; reverse 5′-TCTGGCCTGTATCCAACACTTC-3′) were used. The resulting amplicon sizes were 186 bp, 104 bp and 101 bp, respectively. All primers were obtained from Eurogentec (Belgium). All qPCR samples were loaded in duplicate. Amplifications were performed using QuantStudio™ 3 Real-time PCR System (Applied Biosystems™, Thermo Fisher Scientific, USA). qBase+ software (Biogazelle, Belgium, RRID SCR_003370) was used to identify stably expressed reference genes [57] and was subsequently used for gene normalization. The calculation of relative gene expression was done using the Pfaffl method [58], taking into account primer efficiencies. Sample sizes were calculated *a priori* using G*Power (RRID:SCR_013726) and based on s.d. from previous findings within the research group [57].

### 2.10. Enzyme-linked immunosorbent assay

NMU protein levels were measured in hypothalamus and hippocampus from wild-type and Nmu-Cre knock-in mice using the Mouse NMU ELISA kit (no. abx518493, Abbexa^®^, UK), following the manufacturer's instructions. Briefly, brain tissue was dissected as described in §2.9, homogenized in 0.01 M PBS and centrifuged. Brain homogenate supernatants or standards were added to a 96-well plate pre-coated with an anti-NMU antibody. The reaction is based on a competitive inhibition between the biotin-labelled NMU and the unlabelled NMU on the pre-coated antibody. Therefore, the optical density (OD) is inversely proportional to the NMU amount bound on the plate. The concentration of NMU was calculated from the OD, measured spectrophotometrically at 450 nm.

### 2.11. Statistical analysis

Graphical representations and statistical analyses were performed using GraphPad Prism 8 software (GraphPad Software, Inc., USA, RRID SCR_002798). Data are expressed in columns with dots representing individual values, with the designation of mean with 95% confidence interval. Data were analysed using *t*-tests (unpaired, two-tailed). Significance threshold was set at alpha = 0.05.

## 3. Results

### 3.1. Generation of a Nmu-Cre knock-in mouse model

The Nmu-Cre knock-in mouse was created by the insertion of a Cre-IRES-*Nmu* cassette in frame with the initiation codon of exon 1 of the *Nmu* gene (figure 1*a*,*b*). This targeting strategy with an IRES site generates one single mRNA containing both coding sequences, thus mediating bicistronic translation. As a result, Cre and NMU will be expressed as discrete proteins, Cre expression being regulated by the endogenous *Nmu* promoter. All Nmu-Cre heterozygous knock-in mice obtained were identified by genotyping (figure 1*c*,*d*). Mice were healthy, viable and fertile. The animals displayed no distinct abnormalities or noticeable developmental defects, based on visual assessment of physical appearance, body condition, unprovoked behaviour and brain morphology. However, continuous monitoring is recommended to detect any potential minor phenotype.

### 3.2. Neuroanatomical characterization of ZsGreen1-expressing cells in brain coronal slices from Nmu-Cre:ZsGreen1 mice

**Figure 2.**
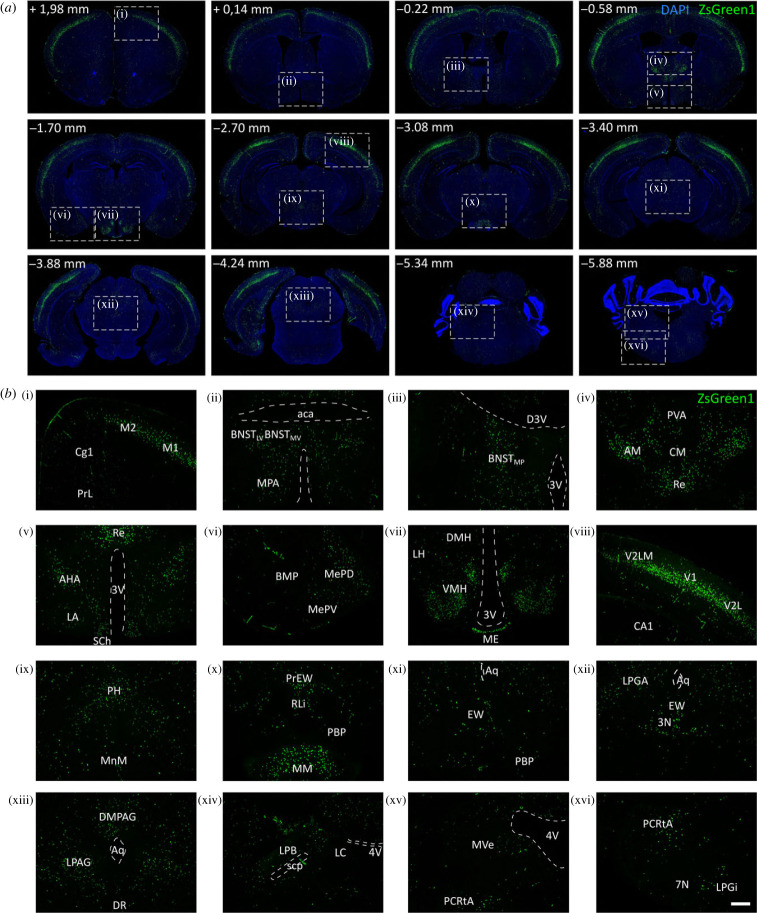
Neuroanatomical distribution of ZsGreen1 expression in coronal brain slices. (*a,b*) Distribution of ZsGreen1-expressing cells in coronal slices from Nmu-Cre:ZsGreen1 mice (representative images from 1 female; *n* = 4 females were analysed). The qualitative analysis was performed on 25–35 coronal slices per mouse brain by two independent observers. DAPI nuclear staining (blue), ZsGreen1 (green). Areas of higher magnification are indicated by a dashed white box. Images were acquired with a fluorescence microscope at 4× (*a*) and 10× (*b*) magnification. Numbers denote neuroanatomical coordinates relative to Bregma. DAPI was used to determine regional boundaries. Scale bar: 300 µm. DAPI: 4′,6-diamidino-2-phenylindole dihydrochloride. Anatomical abbreviations—PrL: prelimbic cortex, Cg1: cingulate cortex area 1, M2: secondary motor cortex, M1: primary motor cortex, aca: anterior commissure, BNST: bed nucleus of stria terminalis (LV) lateroventral part, (MV) medial ventral part, (MP) medial posterior part, MPA: medial preoptic area, 3V: 3rd ventricle (D3V) dorsal, AM: anteromedial thalamic nucleus, PVA: paraventricular thalamic nucleus anterior part, CM: central medial thalamic nucleus, Re: reuniens nucleus, AHA: anterior hypothalamic area, LA: lateroanterior hypothalamic nucleus, SCh: suprachiasmatic nucleus, BMP: basomedial amygdaloid nucleus posterior part, MePD: medial amygdaloid nucleus posterodorsal part (MePV) posteroventral part, LH: lateral hypothalamus, DMH: dorsomedial hypothalamic nucleus, VMH: ventromedial hypothalamic nucleus, ME: medial eminence, CA1: field CA1 of the hippocampus, V2LM: secondary visual cortex lateromedial area, V1: primary visual cortex, V2L: secondary visual cortex lateral area, PH: posterior hypothalamic nucleus, MM: mammillary nucleus (MnM) median part, EW: Edinger–Westphal nucleus (PrEW) pre-nucleus, RLi: rostral linear nucleus of the raphe, PBP: parabrachial-pigmented nucleus of the ventral tegmental area (VTA), Aq: aqueduct, LPGA: lateral periaqueductal grey, 3N: oculomotor nucleus, PAG: periaqueductal grey (DMPAG) dorsomedial part, (LPAG) lateral part, DR: dorsal raphe nucleus, LPB: lateral parabrachial nucleus, scp: superior cerebellar peduncle, LC: locus coeruleus, 4V: 4th ventricle, MVe: medial vestibular nucleus, PCRtA: parvicellular reticular nucleus alpha part, 7N: facial nucleus, LPGi: lateral paragigantocellular nucleus.

Heterozygous Nmu-Cre mice were crossed with homozygous Ai6(RCL-ZsGreen) reporter mice to visualize and characterize NMU-containing cells in the Nmu-Cre mouse model [41] (figure 1*e*,*f*; electronic supplementary material, figure S1). Offspring of this cross were identified by genotyping (figure 1*c*,*d*). Nmu-Cre:ZsGreen1 mice were found to be healthy and viable. To assess the possible spontaneous recombination between LoxP sites and define ZsGreen1 fluorescence background levels, we analysed coronal slices obtained from male and female Ai6 homozygous mice. Minimal and sparse ZsGreen1 signal was observed, consistent with a very low spontaneous recombination rate in the absence of Cre recombinase (electronic supplementary material, figure S2A). Next, coronal slices of both female (figure 2) and male (electronic supplementary material, figure S2B-C) Nmu-Cre:ZsGreen1 mice were systemically analysed by fluorescence microscopy, examining 25 to 35 coronal slices per mouse brain at similar antero-posterior levels. The expression of ZsGreen1 appeared to be stable across litters and consistent between sexes. ZsGreen1 was distributed throughout the cortex, and in subcortical regions comprising the hypothalamus, amygdala, midbrain and brainstem (figure 2*a*,*b*; electronic supplementary material, figures S1 and S2). As previously described, ZsGreen1 fluorescence was mainly detected in the cell bodies and displayed a punctate labelling pattern [41]. Remarkably, ZsGreen1 was photostable, unaffected by fixation, and was readily detected without further amplification [59]. A qualitative gradation was used to describe mainly absent (−), low (+), moderate (++) and high (+++) ZsGreen1 levels (table 3). The nomenclature used in the text, figures and tables follows the mouse brain atlas of Paxinos & Franklin [48]. All the brain regions examined for endogenous expression of ZsGreen1 are listed in table 3 (table 4 for explanation of other abbreviations, anatomical abbreviations are listed). ZsGreen1-expressing cells were observed throughout the cortex, from anterior to posterior levels. While only a few marked cell bodies were detected in the medial prefrontal cortex (mPFC), moderate ZsGreen1 expression levels were found in motor and somatosensory areas, located mainly in layers II/III, IV and V. Remarkably, a high and dense signal was observed in the visual cortex, comprising both primary (V1) and secondary (V2) visual areas. Very low ZsGreen1 expression was found in the nucleus accumbens (NAc), located sparsely in the shell, and dorsally, in the lateral septum (LS), while it was mainly absent in striatal, basal forebrain and hippocampal regions. By contrast, moderate to high ZsGreen1 expression levels were detected in the bed nucleus of stria terminalis (BNST). Similarly, moderate ZsGreen1 expression was clearly observed in a few thalamic nuclei surrounding the paraventricular thalamic nucleus (PV), in the medial amygdaloid nucleus (MeA) and in several nuclei within the hypothalamus. The SCh, VMH and the mammillary body (MM) showed the most robust and dense fluorescent expression. ZsGreen1-marked cells were also observed within the midbrain, especially throughout the periaqueductal grey (PAG). Ventral to the aqueduct, neurons within the Edinger–Westphal nucleus (EW) showed moderate expression, which was also detected more ventrally, in the anterior portion of the rostral linear nucleus of the raphe (RLi) and the parabrachial-pigmented nucleus (PBP) of the ventral tegmental area (VTA). Low to moderate expression of ZsGreen1 was found in regions within the medulla, such as the parvicellular reticular nucleus, alpha part (PCRtA).

**Table 3.** Qualitative analysis of ZsGreen1 fluorescence levels. The qualitative gradation described mainly absent (−), low ( + ), moderate ( + +) and high ( + + + ) ZsGreen1 expression levels. The nomenclature used follows the mouse brain atlas of Paxinos & Franklin [48].

	brain region	present study		previous literature
		ZsGreen1 expression (Nmu-Cre:ZsGreen1 mice)	*Nmu* mRNA (ISH by RNAscope®–chromogenic detection)	*Nmu* mRNA
cortex	medial prefrontal cortex—mPFC	−/+	−/+	
	motor—M1, M2	++	+	
	somatosensory—S1	++	+	
	visual—V1, V2	+++	+	
striatum	caudate putamen— CPu	−		
basal forebrain	lateral septum—LS	+	−/+	
	nucleus accumbens—NAc	−/+		
	ventral pallidum—VP	−		
bed nucleus of stria terminalis	lateral division, ventral part—BNST_LV_	+/++	+	
	lateral division, posterior part—BNST_LP_	−	−	
	medial division, anterior part—BNST_MA_	++	++	
	medial division, posterior part—BNST_MP_	++		
	medial division, ventral part—BNST_MV_	++	++/+++	
hippocampal formation		−/+	−/+	*+++ CA3* (*ISH, Nmu overexpression*) [60]
thalamus	reuniens thalamic nucleus—Re	++/+++	+	+ (RT-qPCR) [10]
	anteromedial thalamic nucleus—AM	++/+++	+	
	central medial thalamic nucleus—CM	+	+	
	paraventricular thalamic nucleus—PV	−/+	+	
amygdala	medial amygdaloid nucleus—MeA	++	+	
	basomedial amygdaloid nucleus—BMA	−/+	−	
	basolateral amygdaloid nucleus—BLA	−/+	−/+	
	central amygdaloid nucleus—CeA	−	−	
hypothalamus	medial preoptic area—MPA	++	+++	detected, not shown (ISH) [26]
	lateroanterior hypothalamic nucleus—LA	++	+	
	anterior hypothalamic area—AHA	++	+	
	suprachiasmatic nucleus—SCh	+++	+++	++ (ISH, not shown) [21]
				+++ (ISH) [26]
				+++ (*ISH, Nmu overexpression*) [60]
	ventromedial hypothalamic nucleus—VMH	+++	++/+++	++ (ISH) [26]
	dorsomedial hypothalamic nucleus—DMH	+	+	+ (ISH, not shown) [21]
				+++ (ISH) [26]
	arcuate nucleus—ARC	+	+	+/++ (ISH) [26]
	lateral hypothalamus—LH	+/++	++	
	medial mammillary nucleus, medial part—MM	++/+++	+/++	detected, not shown (ISH) [26]
	posterior hypothalamic nucleus—PH	++	++	
midbrain	Edinger–Westphal nucleus—EW	++	++	detected, not shown (ISH) [26]
	parabrachial-pigmented nucleus of the ventral tegmental area—PBP	+	++	
	periaqueductal grey—PAG	++	+	
	rostral linear nucleus of the raphe—RLi	+	++	
	substantia nigra—SN	−	−	
	dorsal raphe—DR	−		
hindbrain and medulla	locus coeruleus—LC	−		
	parabrachial nucleus—PB	+		
	parvicellular reticular nucleus, alpha part—PCRtA	+/++	++/+++	
	lateral paragigantocellular nucleus—LPGi	+	+	
	medial vestibular nucleus—MVe	+	+

**Table 4.** List of brain regions and abbreviations. The nomenclature used follows the mouse brain atlas of Paxinos & Franklin [48].

abbreviation	corresponding brain region
3N	oculomotor nucleus
3V	third ventricle
4V	fourth ventricle
7N	facial nucleus
aca	anterior commissure
AHA	anterior hypothalamic area
AM	anteromedial thalamic nucleus
ARC	arcuate nucleus
Aq	aqueduct
BLA	basolateral amygdaloid nucleus
BM	basomedial amygdaloid nucleus
BMP	basomedial amygdaloid nucleus, posterior part
BNST	bed nucleus of the stria terminalis
BNST_LD_	bed nucleus of the stria terminalis, lateral division
BNST_LP_	bed nucleus of the stria terminalis, lateral division, posterior part
BNST_LV_	bed nucleus of the stria terminalis, lateral division, ventral part
BNST_MA_	bed nucleus of the stria terminalis, medial division, anterior part
BNST_MP_	bed nucleus of the stria terminalis, medial division, posterior part
BNST_MV_	bed nucleus of the stria terminalis, medial division, ventral part
CA3	field CA3 of the hippocampus
CeA	central amygdaloid nucleus
Cg1	cingulate cortex, area 1
CM	central medial thalamic nucleus
CPu	caudate putamen
D3V	third ventricle, dorsal part
DG	dentate gyrus
DMH	dorsomedial hypothalamic nucleus
DMPAG	dorsomedial periaqueductal gray
DR	dorsal raphe nucleus
EW	Edinger–Westphal nucleus
Gt	gigantocellular reticular nucleus
GP	globus pallidus
LA	lateroanterior hypothalamic nucleus
LC	locus coeruleus
LH	lateral hypothalamus
LP	lateroposterior thalamic nucleus
LPAG	lateral periaqueductal gray
LPB	lateral parabrachial nucleus
LPGi	lateral paragigantocellular nucleus
LS	lateral septum
LV	lateral ventricle
IF	interfascicular nucleus
IRt	intermediate reticular nucleus
IPR	interpeduncular nucleus, rostral subnucleus
M1	primary motor cortex
M2	secondary motor cortex
MD	mediodorsal thalamic nucleus
ME	medial eminence
MeA	medial amygdaloid nucleus
MePD	medial amygdaloid nucleus, posterodorsal part
MePV	medial amygdaloid nucleus, posteroventral part
MM	medial mammillary nucleus, medial part
MnM	medial mammillary nucleus, median part
MPA	medial preoptic area
MPB	medial parabrachial nucleus
MRe	mammillary recess of the third ventricle
MVe	medial vestibular nucleus
NAc	nucleus accumbens
Opt	optic tract
PAG	periaqueductal gray
PB	parabrachial nucleus
PBP	parabrachial-pigmented nucleus of the ventral tegmental area
PCRtA	parvicellular reticular nucleus, alpha part
PrEW	pre-Edinger–Westphal nucleus
PLH	peduncular part of the lateral hypothalamus
PH	posterior hypothalamic nucleus
PrL	prelimbic cortex
PV	paraventricular thalamic nucleus
PVA	paraventricular thalamic nucleus, anterior part
PVH	paraventricular hypothalamic nucleus
RChl	retrochiasmatic area, lateral part
Re	reuniens thalamic nucleus
RLi	rostral linear nucleus of the raphe
RM	retromammillary nucleus
RMM	retromammillary nucleus, medial part
S1	primary somatosensory cortex
SCh	suprachiasmatic nucleus
Scp	superior cerebellar peduncle
SNC	substantia nigra, compact part
SNR	substantia nigra, reticular part
V1	primary visual cortex
V2	secondary visual cortex
V2L	secondary visual cortex, lateral area
V2ML	secondary visual cortex, mediolateral area
VLPAG	ventrolateral periaqueductal grey
vlVMH	ventrolateral part of the ventromedial hypothalamic nucleus
VMH	ventromedial hypothalamic nucleus
VP	ventral pallidum

### 3.3. Neuroanatomical characterization of ZsGreen1-expressing cells in cleared mouse brains

**Figure 3.**
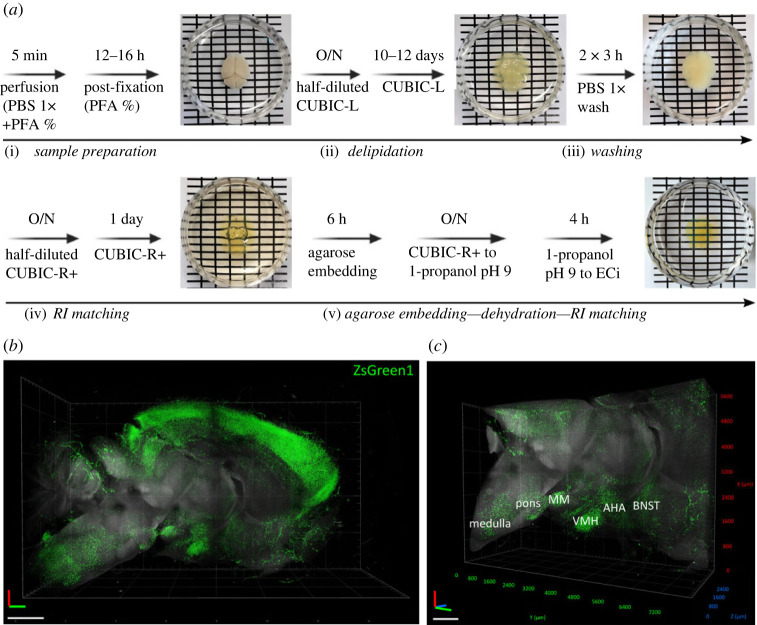
Neuroanatomical distribution of ZsGreen1 expression in cleared mouse brains. (*a*) Clearing and decolouring protocol using CUBIC-L, and refractive index matching protocol using the complementary effect of both CUBIC-R and ECi. After perfusion, postfixed brains (i) were immersed in half-diluted CUBIC-L clearing solution overnight. Lipid removal (ii) was achieved by treating the brain with complete CUBIC-L for 10–12 days, with gentle shaking (75 rpm) at 37°C. The brains were washed in PBS (iii) and then immersed in half-diluted CUBIC-R + (M) O/N and changed to complete CUBIC-R + (M) for a further 24 h (iv). After the first refractive index matching step, cleared brains were embedded in agarose and final matching was achieved by dehydrating the samples in increasing concentrations of 1-propanol pH 9, followed by increasing concentrations of ECi (v). Samples were stored in fresh ECi at room temperature until imaging. (*b*) Overview three-dimensional volume rendering of half brain from a Nmu-Cre:ZsGreen1 mouse and (*c*) XYZ-plane clipping of (*b*). Representative images from 1 mouse brain; *n* = 3 brains were cleared and analysed. Objective 2×, digital zoom 0.63; 488 nm and 561 nm lasers were used to reveal ZsGreen1 endogenous fluorescence (green) and background signal (grey), respectively. Scale bar: 1 mm. PBS: phosphate-buffered saline; PFA: paraformaldehyde; CUBIC: clear unobstracted brain imaging cocktails and computational analysis; ECi: ethyl cinnamate; O/N: overnight. Anatomical abbreviations—MM: mammillary nucleus, VMH: ventromedial hypothalamic nucleus, AHA: anterior hypothalamic area, BNST: bed nucleus of stria terminalis.

To provide a complete picture and a more comprehensive description of ZsGreen1 expression in Nmu-Cre:ZsGreen1 mice, we optimized a new protocol for optimal delipidation, decolouring and refractive index matching based on a combination of existing clearing and RI matching reagents (figure 3*a*). Delipidation and decolouring were performed using CUBIC-L [50,51]. CUBIC-L induced limited swelling, which was minimized further during the first RI matching step with CUBIC-R + (M) [51,52]. After dehydration with pH-adjusted 1-propanol series, a last step using the organic solvent ECi was performed, taking advantage of the complementary RI matching effect of both CUBIC-R and ECi [55]. The combination of 1-propanol pH 9 and ECi preserved endogenous fluorescence [54–56]. In the optically cleared tissue, we performed volumetric imaging of ZsGreen1 signal using LSFM. This revealed a continuous population of ZsGreen1-expressing cells extending from the ventral forebrain to the caudal midbrain, comprising numerous regions such as the ST, medial preoptic area (MPA), SCh, VMH, lateral hypothalamus (LH), MeA, posterior hypothalamus (PH), MM, EW, VTA and PAG (figure 3*b*,*c*; electronic supplementary material, video S1). ZsGreen1 expression extends beyond the midbrain, through the pons and the medulla (figure 3*b*,*c*; electronic supplementary material, video S1). Therefore, this protocol provides optimal clearing of mouse brain while successfully preserving the endogenous ZsGreen1 fluorescence. The observed distribution pattern was consistent with discrete coronal slices.

### 3.4. Validation of the Nmu-Cre knock-in mouse model

The ZsGreen1 distribution pattern observed in our study was more widespread than anticipated based on the literature, although it is comparable with the distribution observed in the Nmu-Cre BAC transgenic mice [42]. It is known that transient developmental expression of Cre can contribute residual fluorescent expression in cells where the *Nmu* promoter is no longer active in adult life [40]. We therefore carried out additional validation experiments.

#### 3.4.1. Reverse-transcription quantitative polymerase chain reaction and enzyme-linked immunosorbent assay

**Figure 4.**
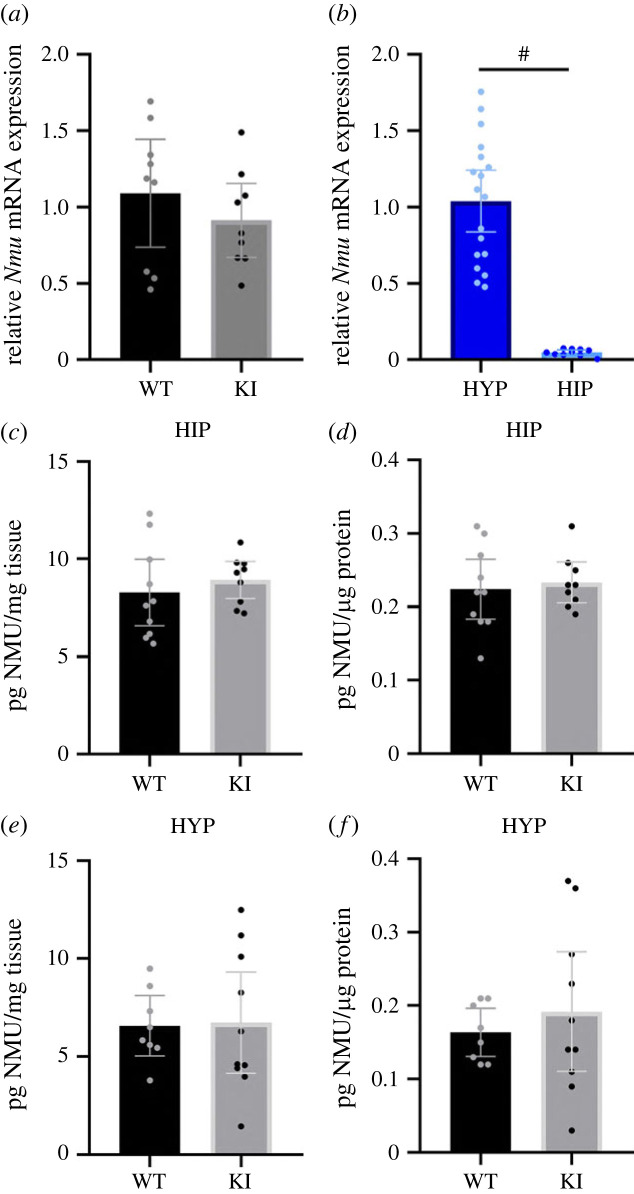
Quantification of the expression of *Nmu* mRNA and NMU protein by using RT-qPCR and ELISA. (*a*) Relative *Nmu* mRNA expression in the hypothalamus of wild-type (*n* = 9) and Nmu-Cre knock-in mice (*n* = 9). (*b*) The comparison of *Nmu* mRNA expression in the hypothalamus (*n* = 18) and the hippocampus (*n* = 10), considering results obtained from both wild-type and Nmu-Cre mice. In the case of the hippocampus, nine samples (three from wild-type and five from Nmu-Cre mice) were not considered in the analysis due to non-specific amplification or primer-dimers as reflected in multiple peaks in the melt curve (Ct above 30). The experiment was replicated two times. Data were analysed with the Pfaffl method and normalized to the reference genes *B2m* and Hprt1. Dots represent individual data points; representation of the mean with a 95% confidence interval. Data were analysed by student's *t*-test; ^#^*p* < 0.0001. (*c–f*) NMU protein levels in the hippocampus (*c,d*) of wild-type (*n* = 10) and Nmu-Cre knock-in mice (*n* = 9), and in the hypothalamus (*e,f*) of wild-type (*n* = 8) and Nmu-Cre knock-in mice (*n* = 10), expressed as pg NMU per mg tissue (*c,e*) or pg NMU per µg protein (*d,f*). Dots represent individual data points; representation of the mean with a 95% confidence interval. WT: wild-type; KI: knock-in; HYP: hypothalamus; HIP: hippocampus.

The design of the Nmu-Cre knock-in mouse model was based on the absence of regulatory elements that may be impacted by the insertion of the Cre-IRES-*Nmu*-hGHpolyA recombination cassette in exon 1 of the *Nmu* gene. To assess whether the Nmu-Cre knock-in mouse model expressed *Nmu* and did so at similar levels to those observed in wild-type mice, RT-qPCR analyses were carried out. We selected the hypothalamus and hippocampus to assess *Nmu* transcript levels between wild-type and Nmu-Cre knock-in mice and test for regional differences, as described in the literature and consistently in Nmu-Cre:ZsGreen1 mice. RT-qPCR results, normalized to the expression of the reference genes *B2m* and *Hprt1*, showed similar relative *Nmu* mRNA expression levels in the hypothalamus of both Nmu-Cre and wild-type mice, indicating that *Nmu* mRNA is indeed expressed in the Nmu-Cre mouse and this expression is not significantly dysregulated in the knock-in mouse model (figure 4*a*). To further examine whether ZsGreen1 signal represented the endogenous NMU expression pattern, relative *Nmu* mRNA expression levels were compared between hypothalamus and hippocampus. The results did indeed show significantly higher levels in hypothalamus compared to those found in the hippocampus (figure 4*b*). Similarly, the NMU protein content in the hypothalamus and hippocampus was measured using an enzyme-linked immunosorbent assay (ELISA) to verify that protein levels were unaltered in Nmu-Cre knock-in mice compared to wild-type mice. The results showed similar NMU protein concentration in both brain regions of Nmu-Cre knock-in and wild-type mice (figure 4*c–f*).

#### 3.4.2. *In situ* hybridization

**Figure 5.**
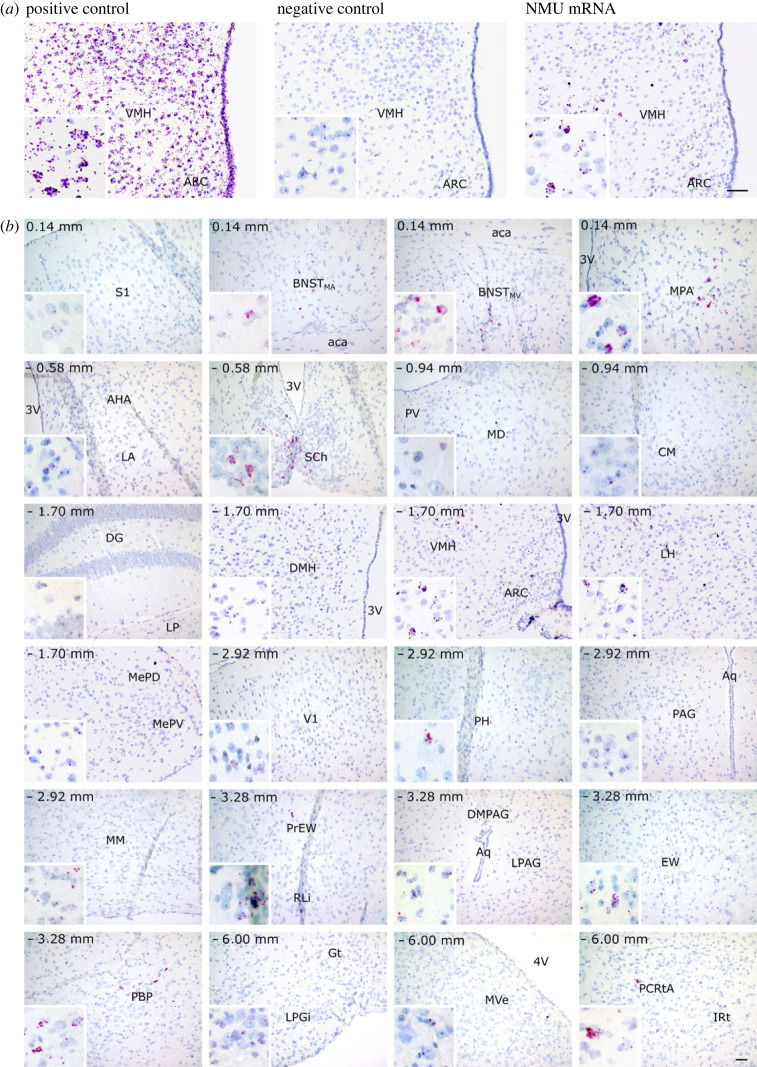
*Nmu* mRNA expression pattern analysis in Nmu-Cre knock-in mice by *in situ* hybridization (RNAscope®). (*a*) Representative images of a positive control (RNAscope® probe against the *Mus musculus* peptidyl-prolyl isomerase B (Ppib); left), negative control (RNAscope® probe against the *Bacillus subtilis* dihydrodipicolinate reductase (dapB); middle) and *Nmu* mRNA (right). Haematoxylin nuclear staining (blue); *Nmu* mRNA signal (red). (*b*) *Nmu* mRNA expression pattern in coronal sections from Nmu-Cre mice. Areas of higher magnification are outlined in white (left-bottom). Representative images from 1 mouse brain; *n* = 2 brains were analysed. Images were acquired with the brightfield microscope Olympus BX61 at 20× and 40× magnification. Haematoxylin was used to determine regional boundaries. Scale bar: 100 µm. Anatomical abbreviations—VMH: ventromedial hypothalamic nucleus, ARC: arcuate nucleus, S1: primary somatosensory cortex, aca: anterior commissure, BNST: bed nucleus of stria terminalis (MA) medial anterior part, (MV) medial ventral part, 3V: 3rd ventricle, MPA: medial preoptic area, AHA: anterior hypothalamic area, LA: lateroanterior hypothalamic nucleus, SCh: suprachiasmatic nucleus, PV: paraventricular thalamic nucleus, MD: mediodorsal thalamic nucleus, CM: central medial thalamic nucleus, DG: dentate gyrus, LP: lateroposterior thalamic nucleus, DMH: dorsomedial hypothalamic nucleus, LH: lateral hypothalamus, MePD: medial amygdaloid nucleus posterodorsal part (MePV) posteroventral part, V1: primary visual cortex, PH: posterior hypothalamic nucleus, PAG: periaqueductal grey (DMPAG) dorsomedial part, (LPAG) lateral part, Aq: aqueduct, MM: mammillary nucleus, EW: Edinger–Westphal nucleus (PrEW) pre-nucleus, RLi: rostral linear nucleus of the raphe, PBP: parabrachial-pigmented nucleus of the ventral tegmental area (VTA), MVe: medial vestibular nucleus, PCRtA: parvicellular reticular nucleus alpha part, LPGi: lateral paragigantocellular nucleus, Gt: gigantocellular reticular nucleus, IRt: intermediate reticular nucleus, 4V: 4th ventricle.

To further assess whether ZsGreen1-expressing cells faithfully represented endogenous NMU expression pattern, *Nmu* mRNA expression was analysed by RNA ISH using RNAscope^®^ technology, a method that provides higher sensitivity and specificity for the detection of low-abundant transcripts than classical ISH techniques [34]. Indeed, while we failed to detect *Nmu* transcript using classical FISH, we did obtain reliable results using RNAscope^®^. Initial attempts to detect *Nmu* mRNA using fluorescent probes and its overlap with ZsGreen1 signal in brain coronal slices from Nmu-Cre:ZsGreen1 mice were unsuccessful. The RNAscope^®^ *in situ* signal detected was unconsistent between mice, and sample processing during the ISH protocol resulted in an almost complete loss of ZsGreen1 fluorescent signal, which prevented us from verifying whether the RNAscope^®^ signal overlapped with the green fluorescent signal. In addition, the lack of suitable anti-ZsGreen1 antibodies hampered the detection of ZsGreen1 after the ISH procedure. Further attempts using RNAscope^®^ and chromogenic detection in Nmu-Cre mice showed consistent results (figure 5). Each punctate dot signal represents a single *Nmu* mRNA molecule while clusters result from multiple mRNA molecules, visualized in red in contrast with nuclei stained with haematoxylin. Overall, the ISH results in Nmu-Cre mice are largely in agreement with the ZsGreen1 results found in Nmu-Cre:ZsGreen1 mice (table 3). However, the expression of *Nmu* mRNA was found to be generally lower and more restricted. In line with the ZsGreen1 data, moderate to high *Nmu* mRNA signal was found in several divisions of the BNST, as well as in hypothalamic, midbrain and hindbrain nuclei. The most robust transcript signal was found in the SCh, MPA and VMH. By contrast, very low *Nmu* mRNA expression was observed through the cortex, amygdala and thalamus, where a strong ZsGreen1 signal was described. Notably, only a few cells expressing very low amounts of transcript were detected in visual areas, where a moderate and consistent ZsGreen1 signal was observed.

### 3.5. Identification of the cell-type-expressing neuromedin U

**Figure 6.**
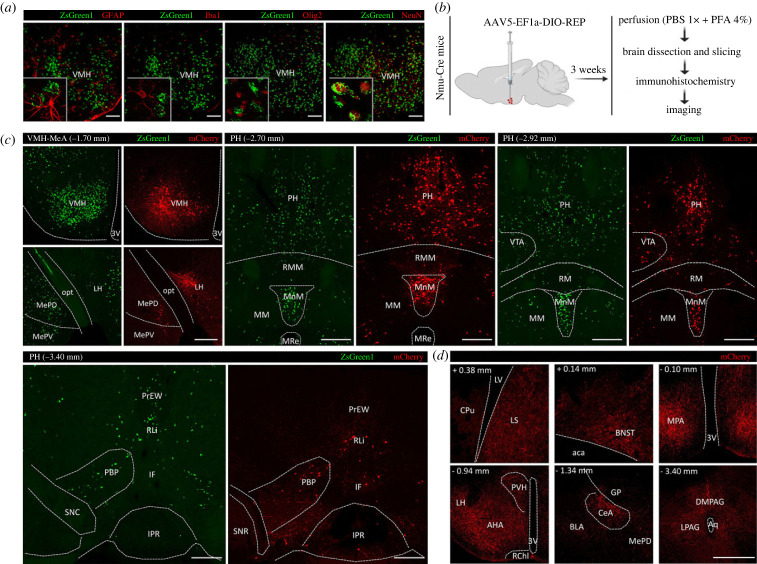
Characterization of NMU neurons in the VMH using IHC and a viral tracer. (*a*) Immunohistochemical analysis of brain coronal slices from Nmu-Cre:ZsGreen1 mice at the level of the VMH. In green, ZsGreen1 fluorescence; in red, GFAP and Olig2 (secondary antibodies coupled to Cy3), NeuN and Iba1 (secondary antibodies coupled to Cy5). Representative images from 1 mouse brain; *n* = 3 brains were analysed. Images were acquired with a confocal microscope with a 20× objective lens. Areas of higher magnification are outlined in grey (left-bottom). Scale bar: 100 µm. GFAP: glial fibrillary acidic protein; Iba1: ionized calcium-binding adaptor molecule 1; Olig2: oligodendrocyte transcription factor 2; NeuN: neuronal nuclear protein. (*b*) Schematic representation of the intracerebral injection of the viral tracer AAV5-EF1a-DIO-RFP into the VMH of Nmu-Cre mice. Three weeks after surgery, mice were transcardially perfused, and brains were processed for immunohistochemical amplification of the RFP fluorescent signal with an anti-mCherry primary antibody and a Cy3-conjugated secondary antibody (red). (*c*) Representative images from coronal brain slices showing a comparison of Cre-dependent RFP expression (red) after the viral vector infusion into the VMH of Nmu-Cre mice, and endogenous ZsGreen1 expression (green) in Nmu-Cre:ZsGreen1 mice. The extent of transduction was determined by RFP fluorescence, analysed in consecutive slices from −0.94 mm to −2.30 mm. The boundaries of the VMH were determined using DAPI (not shown), considering the size and ventral shape of the third ventricle and the different density of cells that characterize the ARC, LH and DMH. ImageJ (*BigWarp* plugin) was used to superimpose the images with the boundaries defined in the Paxinos & Franklin Atlas [48]. (*d*) Representative images from coronal brain slices showing neuronal projections from VMH^NMU^ neurons after the viral vector infusion into the VMH of Nmu-Cre mice. Representative images from 1 mouse brain; *n* = 3 brains were analysed. Images from (*c*) and (*d*) were acquired with a confocal microscope with a 10× objective lens. Scale bar: 300 µm. Anatomical abbreviations—VMH: ventromedial hypothalamic nucleus, 3V: 3rd ventricle, MePD: medial amygdaloid nucleus posterodorsal part (MePV) posteroventral part, opt: optic tract, LH: lateral hypothalamus, PH: posterior hypothalamic nucleus, RM: retromammillary nucleus (RMM) medial part, MM: mammillary nucleus (MnM) median part, MRe: mammillary recess of the 3rd ventricle, VTA: ventral tegmental area, EW: Edinger–Westphal nucleus (PrEW) pre-nucleus, RLi: rostral linear nucleus of the raphe, PBP: parabrachial-pigmented nucleus of the (VTA), IF: interfascicular nucleus, SNC: substantia nigra compact part (SNR) reticular part, IPR: interpeduncular nucleus rostral subnucleus, LV: lateral ventricle, CPu: caudate putamen, LS: lateral septum, AHA: anterior hypothalamic area, LH: lateral hypothalamus, PVH: paraventricular hypothalamic nucleus, 3V: 3rd ventricle, BNST: bed nucleus of stria terminalis, aca: anterior commissure anterior part, RChl: retrochiasmatic area lateral part, GP: globus pallidus, CeA: central amygdaloid nucleus, BLA: basolateral amygdaloid nucleus, MPA: medial preoptic area, Aq: aqueduct, PAG: periaqueductal grey (LPAG) lateral part, (DMPAG) dorsomedial part.

**Figure 7.**
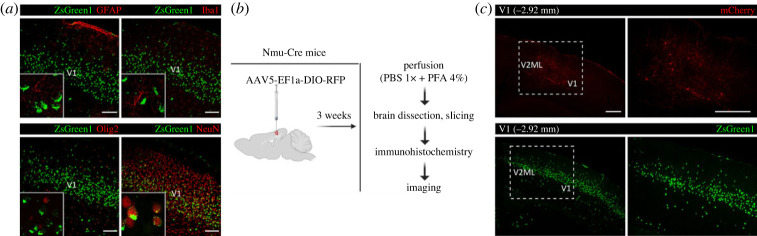
Characterization of NMU neurons in the primary visual cortex (V1) using IHC and a viral tracer. (*a*) Immunohistochemical analysis of brain coronal slices from Nmu-Cre:ZsGreen1 mice at the level of the V1. In green, ZsGreen1 fluorescence; in red, GFAP and Olig2 (secondary antibodies coupled to Cy3), NeuN and Iba1 (secondary antibodies coupled to Cy5). Representative images from 1 mouse brain; *n* = 3 brains were analysed. Images were acquired with a confocal microscope at 20× magnification. Areas of higher magnification (40×) are outlined in grey (left-bottom). Scale bar: 100 µm. GFAP: glial fibrillary acidic protein; Iba1: ionized calcium-binding adaptor molecule 1; Olig2: oligodendrocyte transcription factor 2; NeuN: neuronal nuclear protein. (*b*) Schematic representation of the intracerebral injection of the viral tracer AAV5-EF1a-DIO-RFP into the V1 of Nmu-Cre mice. Three weeks after surgery, mice were transcardially perfused, and brains were processed for immunohistochemical amplification of the RFP fluorescent signal with an anti-mCherry primary antibody and a Cy3-conjugated secondary antibody (red). (*c*) Representative images from coronal brain slices showing a comparison of Cre-dependent RFP expression (red) after the viral vector infusion into the V1 of Nmu-Cre mice, and endogenous ZsGreen1 expression (green) in Nmu-Cre:ZsGreen1 mice. Areas of higher magnification are indicated by a dashed white box. DAPI (not shown) was used to determine regional boundaries. Representative images from 1 mouse brain; *n* = 2 brains were analysed. Images were acquired with a confocal microscope. Scale bar: 300 µm. Anatomical abbreviations—V1: primary visual cortex, V2ML: secondary visual cortex mediolateral area.

Nmu-Cre:ZsGreen1 mice were used to identify the molecular identity of NMU-expressing cells. Immunostainings using antibodies against glial fibrillary acidic protein (GFAP), ionized calcium-binding adaptor molecule 1 (Iba1), oligodendrocyte transcription factor 2 (Olig2) and neuronal nuclear protein (NeuN) were used as markers for astrocytes, microglia, oligodendrocytes and neurons, respectively. The analyses of colocalization between these specific markers and the fluorescent protein ZsGreen1 were performed in the VMH (figure 6*a*) and visual cortex (figure 7*a*) of coronal slices using confocal microscopy. As hypothesized, ZsGreen1-expressing cells showed a general overlap with the neuronal marker NeuN but not with the glial markers GFAP, Iba1 and Olig2. These results confirm that NMU-expressing cells are mainly neurons.

### 3.6. Neuroanatomical characterization of neuromedin U-expressing cells in the ventromedial hypothalamic nucleus and visual cortex

Consistent with the literature [26], we found robust expression of ZsGreen1 in the VMH, consistently expressing moderate to high levels of *Nmu* transcript. Therefore, we selected the VMH to further characterize NMU-expressing neurons (VMH^NMU^). We performed intracerebral injections of the double-floxed AAV5-EF1a-DIO-RFP viral tracer into the VMH of Nmu-Cre mice, to drive Cre-dependent expression of the RFP (figure 6*b*). By infusing the viral tracer into the VMH, RFP expression into the target region allowed us to further confirm that Cre recombinase is indeed expressed and still active in the VMH of adult Nmu-Cre mice (figure 6*c*). In addition, taking advantage of the anterograde and retrograde axonal transport ability reported for the AAV5 serotype [61–63], we described input regions to the VMH^NMU^ (figure 6*c*) and output regions receiving projections from the VMH^NMU^ (figure 6*d*). A detailed analysis of RFP expression showed RFP-marked cell bodies in the LH and MeA, and in posterior hypothalamic and midbrain regions such as the MM, PH and PBP, and to a lesser extent in the EW and RLi (figure 6*c*), regions where ZsGreen1 expression was described. Terminals from the VMH^NMU^ were found in anterior regions, at the level of the LS and ST, and within the hypothalamus, including the MPA, anterior hypothalamic area (AHA), LH, DMH and paraventricular hypothalamic nucleus (PVH). Projections were also found to the central amygdaloid nucleus (CeA), and to posterior regions including the PAG and, to a lesser extent, the EW (figure 6*d*). In addition to the VMH, a clear cluster of ZsGreen1-expressing cells was observed in the visual cortex. This is in contrast with the literature [10,21,26] and our ISH data that showed a few cells expressing low amount of *Nmu* transcript within the visual cortex. To test whether the *Nmu* promoter was still active in the adult visual cortex, AAV5-EF1a-DIO-RFP was infused in the V1 (figure 7*b*), revealing RFP-marked cells in the target region (figure 7*c*). The RFP expression pattern was similar to that described for ZsGreen1; however, only a few RFP-marked cells were observed, in contrast with the strong ZsGreen1 signal but in line with the mRNA ISH results obtained in visual areas.

### 3.7. Evaluation of the presence of fast-acting neurotransmitters in neuromedin U neurons

**Figure 8.**
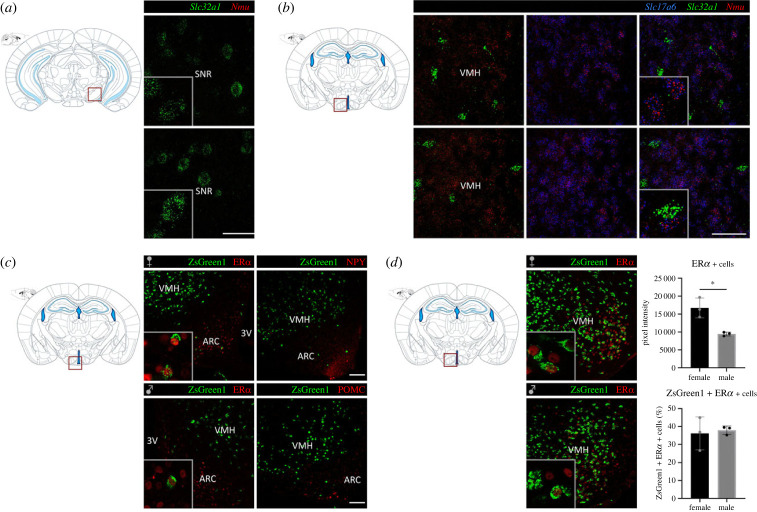
Characterization of NMU neurons in the VMH using IHC and single-molecule fluorescent *in situ* hybridization (RNAscope®). (*a*) RNAscope® for *Slc32a1* (green, VIAAT) and *Nmu* (red) mRNA in the SNR showing the expression of the vesicular transporter VIAAT but failing to detect *Nmu* mRNA. (*b*) RNAscope® for *Slc17a6* (blue, VGLU2), *Slc32a1* (green, VIAAT) and *Nmu* (red) mRNA in the VMH. Representative images from 1 mouse brain; *n* = 2 brains were analysed. Images were acquired with a confocal microscope at 40× magnification. Areas of higher magnification are outlined in grey (left-bottom). Scale bar: 50 µm. (*c*) Analysis of ZsGreen1 co-expression ER*α*. Immunohistochemical analysis of brain coronal slices from Nmu-Cre:ZsGreen1 male and female mice at the level of the VMH. In green, endogenous ZsGreen1 fluorescence; in red, ER*α* (secondary antibody coupled to Cy5). Overlap of ZsGreen1 and ER*α* in the ventrolateral VMH was quantified using ImageJ (*n* = 3 males and *n* = 3 females). Graphs indicating ER*α* signal measured as pixel intensity (top) and % of ZsGreen1 positive cells expressing ER*α* (bottom). Data were analysed by student's *t*-test; *p < 0.05. (*d*) Analysis of ZsGreen1 co-expression with ER*α*, NPY and POMC. Immunohistochemical analysis of brain coronal slices from Nmu-Cre:ZsGreen1 male and female mice at the level of the VMH and arcuate nucleus (ARC). In green, endogenous ZsGreen1 fluorescence; in red, ER*α*, NPY and POMC (secondary antibody coupled to Cy5). Images of (*c*) and (*d*) were acquired with a confocal microscope at 20× magnification. Areas of higher magnification (40×) are outlined in grey (left-bottom). Scale bar: 100 µm. Schematic representations of coronal slices with the region of interest outlined in red are depicted for (*a*–*d*). DAPI (not shown) was used to determine regional boundaries. VIAAT: vesicular inhibitory amino acid transporter; VGLU2: vesicular glutamate transporter 2; Nmu: neuromedin U; ER*α*: estrogen receptor alpha; NPY: neuropeptide Y; POMC: proopiomelanocortin. Anatomical abbreviations—SNR: substantia nigra reticular part, VMH: ventromedial hypothalamic nucleus, ARC: arcuate nucleus, 3V: 3rd ventricle.

To further characterize VMH^NMU^ neurons, we addressed co-expression of *Nmu* mRNA with markers of the primary excitatory and inhibitory neurotransmitters of the CNS, glutamate and gamma-aminobutyric acid (GABA). Single-molecule fluorescence ISH by RNAscope^®^ was used in coronal brain slices from Nmu-Cre mice for the examination of the targeted RNA within intact cells in the VMH, a region that showed robust *Nmu* mRNA expression. Indeed, fluorescent signal revealing *Nmu* mRNA was found in cells in the VMH, mainly in but also away from the soma, in axons and/or dendrites (figure 8*b*) but was not found in the substantia nigra (SN) (figure 8*a*), a region where, consistent with previous literature [10,21,26], neither ZsGreen1 nor *Nmu* mRNA by chromogenic RNAscope^®^ was detected. In the VMH, expressing mainly glutamatergic cells, *Nmu* mRNA signal was found to overlap with *Slc17a6,* the gene encoding for the vesicular glutamate transporter (VGLU2) but not with *Slc32a1* encoding vesicular inhibitory amino acid (VIAAT; figure 8*b*). However, since the observed expression of *Nmu* mRNA was not restricted to the soma of cells in the VMH, *Nmu* expression in GABAergic neurons cannot be excluded. Additionally, we addressed possible co-expression of *Nmu* mRNA and transcripts of the fast-acting neurotransmitters in another region expressing higher number of GABAergic neurons, such as the anterior BNST. In the BNST, that contains mostly GABAergic neurons with a minority of scattered glutamatergic neurons, *Nmu* mRNA was found both in glutamatergic cells and in cells expressing the gene encoding for the VIAAT (*Slc32a1*) (electronic supplementary material, figure S4). Overall, these results suggest that NMU neurons are not obligate peptidergic neurons, but co-express fast-acting neurotransmitters.

### 3.8. Evaluation of ZsGreen1 co-expression with specific markers in the ventromedial hypothalamic nucleus and arcuate nucleus

Additional immunofluorescence analysis was performed to further characterize VMH^NMU^ neurons in coronal brain slices from Nmu-Cre:ZsGreen1 mice (figure 8*c*,*d*). Robust expression of ZsGreen1 was observed in the VMH, while only a few marked cells were shown in the ARC. This expression was consistent with the ISH data in Nmu-Cre mice. Antibodies against POMC and neuropeptide-Y (NPY) were used as markers of the ARC, while estrogen receptor alpha (ER*α*) was used as a marker of both the ARC and the VMH. Interestingly, the analysis revealed no overlap with POMC-expressing cells nor NPY-expressing cells (figure 8*c*), while fewer than 40% of ZsGreen1 cells colocalized with ER*α* in the ventrolateral VMH (vlVMH) of both male and female adult mice (figure 8*d*). These results suggest that NMU neurons mainly constitute a distinct population of hypothalamic cells.

## 4. Discussion

In the present study, we generated a Nmu-Cre knock-in mouse model constitutively expressing Cre recombinase in all NMU-producing cells. We validated the model and carried out a complete characterization of NMU expression in both male and female adult mouse brain. Additionally, we further characterized NMU neurons in the VMH using a viral tracer, IHC and ISH analysis.

The strategy that was used to generate the Nmu-Cre knock-in mouse model was based on the absence of regulatory elements that may be impacted by the insertion of the Cre-IRES-*Nmu*-hGHpolyA recombination cassette. Selecting the hypothalamus as the NMU-expressing region, as consistently reported in rodents and humans [10,16,19,21,25,26,32] and in agreement with this study, our RT-qPCR analysis indicated that the strategy chosen to generate the knock-in mouse model was not interfering with endogenous *Nmu* expression. In the same line, we found comparable NMU protein levels in both wild-type and Nmu-Cre mice. Our RT-qPCR data also highlighted the regional differences reported previously in the literature [1], comparing the transcript levels between the hypothalamus and the hippocampus. These results were consistent with the clear ZsGreen1 regional differences observed in Nmu-Cre:ZsGreen1 mice, and thus supporting the validation of the model.

Gene targeting is considered the most reliable approach to faithfully mimic endogenous gene expression [38], with the binary system Cre/LoxP being one of the most extensively used strategies for cell-type-specific genetic targeting [38,40,41]. The popularity of this strategy is reflected in the fact that currently anatomical information is available for more than 200 Cre driver lines in the Transgenic characterization database from the Allen Institute for Brain Science [64]. When using Cre driver lines, and after verifying that the genetic strategy does not interfere with the endogenous expression of the target gene, the main question is how faithfully Cre expression represents endogenous gene expression patterns. First, one of the main strategies to demonstrate that Cre recombination occurs in a Cre mouse line is crossing it with a reporter line that carries a Cre-dependent fluorescent reporter gene. The fidelity of Cre expression can be confirmed by comparing the reporter levels with existing literature and, directly, with the protein and/or mRNA expression of the gene of interest. However, the validation of our Nmu-Cre knock-in mouse model was challenging and hampered by the lack of detailed neuroanatomical information in mouse available in the literature, the lack of suitable anti-NMU antibodies and the poor sensitivity of classical mRNA ISH techniques. As such, our present study can be seen as a pioneering study performed with state-of-the-art technologies that brings new insights to the field about the NMU population of neurons in the brain.

In general, our results suggest that the pattern of the observed ZsGreen1 signal broadly represents the endogenous NMU expression. However, the intensity and extent of the transcript signal did not always correlate with the ZsGreen1 signal. ZsGreen1 and *Nmu* transcript expression levels were consistent in several BNST divisions and within the hypothalamic, midbrain, hindbrain and medullary regions analysed. Our data are in agreement with previous literature specifically in certain hypothalamic (SCh, MPA, VMH, MM) and midbrain (EW) regions [10,21,26]. The SCh is the main region where moderate expression of NMU has been consistently described in both rats and mice [21,26,29]. Our results confirm the expression of NMU in this region, in line with the suggested role of the peptide in the regulation of the circadian oscillator system [4]. Remarkably, the neuropeptide neuromedin S (NMS), whose precursor shares high structural similarity with that of NMU, is considered another endogenous ligand of NMUR1 and NMUR2 and has a more restricted expression in the SCh [65]. Indeed, both NMU and NMS have been implicated in the regulation of circadian rhythms [66]. Interestingly, a diurnal rhythm has also been described in the EW [67], supporting the role of NMU in the regulation of the circadian clock. We also found low to moderate levels of both ZsGreen1 and *Nmu* mRNA in the DMH, a region where previous studies showed discrepancies in terms of transcript expression levels [21,26]. In the ARC, we similarly detected very few ZsGreen1 cells and low *Nmu* mRNA levels, which contrasts with the moderate *Nmu* mRNA expression reported in mice by Graham *et al*. [26]. However, probe sensitivity could be a possible explanation for these discrepancies.

We observed some inconsistencies between ZsGreen1 and *Nmu* transcript levels. We detected very low transcript levels of *Nmu* but high ZsGreen1 signal in the cortex (notably the V1 and V2), thalamus (especially in the reuniens thalamic nucleus (Re) and the anteromedial thalamic nucleus (AM)), PAG and MeA. Funes *et al*. [10] used RT-qPCR and reported low levels of *Nmu* transcript in the thalamus, similarly to our ISH data. To the best of our knowledge, neither NMU nor *Nmu* mRNA were previously reported in the mouse cortex, PAG or MeA. Interestingly, cortical NMU expression has been described in humans [68], but expression in rats is still under debate. One study found NMU-LI in pyramidal cells of the cerebral cortex located in the layer V of the somatosensory and motor area [20]. However, these results differed from the study of Ballesta *et al*. [16], who did not detect immunoreactive fibres or terminals in the telencephalon. In our present study, the infusion of an AAV vector specifically driving Cre-dependent expression of the fluorescent protein RFP into the visual cortex showed the presence of very few RFP-marked cells. This confirmed our ISH data and indicated that there is indeed Cre expression in the adult cortex, but in very low levels. Interestingly, transgenic overexpression of NMU in mice showed ubiquitous *Nmu* mRNA expression levels compared to wild-type mice, including remarkable expression throughout the cortex [60]. These results support our data showing cortical expression of NMU, pointing to the need to discuss the biological relevance in terms of amount and role of NMU protein in cortical areas under physiological conditions in adult life.

It is not unusual to find the expression pattern of Cre and Cre reporters more widespread than initially expected [69,70]. In our study, the lack of correlation between the moderate to high ZsGreen1 expression consistently reported throughout the cortex, in certain thalamic and amygdaloid regions, and the very low *Nmu* mRNA levels observed in those regions may be due to a combination of events. First, transient Cre expression during development must be considered. Many transgenes are sensitive to epigenetic regulation and seem to be transiently expressed in cells during the developmental stage but are no longer expressed in adult life. Second, NMU may similarly be expressed in certain regions at early stages of development but silenced at later stages. To our knowledge, no detailed information has been published on the time course of NMU expression in the CNS at different stages of development, but this possibility needs to be considered. Third, even though knock-in mice bypass the positional effect due to random integration, potential ectopic expression of Cre must be contemplated. In our present study, the transient expression of NMU and Cre at early developmental stages could result in unanticipated ZsGreen1 expression in offspring of the heterozygous Nmu-Cre and homozygous Ai6 cross, in cells that no longer express NMU in the adult life. ZsGreen1 half-life has been suggested to be at least 26 h in mammalian cells or slightly longer due to its tetrameric structure, in contrast with the monomeric configuration of other well-known fluorescent proteins [59,71]. Thus, a significant accumulation of fluorescent signal until adult life is not expected. In turn, ectopic expression of Cre could result in unanticipated reporter expression. It is known that the efficiency of the recombination depends, in part, on the location of the LoxP sites in the genome and the distance separating them [72,73]. The distance between both LoxP sites in the Ai6 mice may lead to potential spontaneous recombination, resulting in ZsGreen1 expression in cells that do not express Cre recombinase [41]. However, since the unanticipated expression due to spontaneous recombination is expected to be variable in offspring from the same line, several mice need to be examined in order to identify the effects of these unwanted recombination events [70]. In our study, ZsGreen1 signal was analysed in several male and female adult mice, showing consistent results. It is also important to highlight that every floxed allele can have a different sensitivity to Cre-mediated recombination; this means that different reporter lines can show different Cre activity [72]. Therefore, it may be interesting to cross the new Cre recombinase line with a different reporter line and to examine in detail reporter expression in certain areas at different developmental stages. Ultimately, even though a significant expression of Cre in adult life due to transient and ectopic expression is not expected, the resulting low Cre expression may be sufficient to induce recombination events between LoxP sites, thus generating unintended, but strong and consistent fluorescent signal [70]. Overall, our results suggest that the ZsGreen1 reporter is indeed a useful tool that reveals which regions express NMU in the brain, but these results cannot be extrapolated to the amount of protein that is actually expressed.

Reporter expression is considered to reflect the end result of the complex modulatory activities of transcription and translation. In addition, the lack of suitable anti-NMU antibodies prevents a direct detection of the NMU protein, hampering a comparison with ZsGreen1 and *Nmu* mRNA. It is important to highlight that in the present study we did not examine *Nmu* mRNA expression across different time points. Certainly, *Nmu* mRNA expression was shown to be differentially modulated in different conditions. Like many other neuropeptides, *Nmu* mRNA expression also exhibits a circadian rhythm in the SCh. In rats, *Nmu* mRNA levels peak between circadian times 4 and 8, which represents the subjective day [29] and have also been shown to fluctuate during post-natal maturation and oestrus cycle [2]. Therefore, it may be interesting to use short half-life Cre reporter mice for a longitudinal monitoring of the *Nmu* transcriptional activity in future work. Even though the correlation between mRNA expression and protein expression levels can be relatively low, the detection of the mRNA is generally considered a good indicator of the presence of a protein [74], and a better approximation of the amount of protein present than the reporter expression. Overall, the NMU distribution revealed by our ZsGreen1 data and ISH results confirms and significantly extends the current knowledge on NMU distribution in the mouse brain.

The description of the distribution of ZsGreen1-expressing cells was performed not only in discrete coronal slices but in the whole brain. The combination of optical tissue clearing and LSFM enabled us to reach a better understanding of the neuroanatomical distribution of NMU-expressing cells in the adult mouse brain, since it provides a complete picture in intact tissue. The protocol optimized in this study represents a novel option for tissue clearing, combining the powerful and effective CUBIC clearing protocol with the complementary refractive index matching of CUBIC-R and ECi to obtain optimally optical cleared tissue. The analysis unveiled a continuous population of ZsGreen1-expressing cells extending from the ventral forebrain to the caudal midbrain, and beyond the midbrain through the pons and the medulla. It is tempting to speculate on the existence of a midline circuit of NMU-expressing regions projecting to and receiving inputs from other NMU-expressing regions. Indeed, the results of the infusion of the double-floxed AAV5-EF1a-DIO-RFP viral tracer into the VMH suggest this possibility. The bidirectional axonal transport ability of the AAV5 serotype has been observed when injected into the CNS in both mouse and rat [61–63], while it did not exhibit anterograde transneuronal transport [75]. Thus, the detection of marked cell bodies in distant areas from the target region strongly suggests retrograde transport of the viral tracer and transduction of NMU-expressing neurons. Our results revealed the existence of NMU-expressing neurons in hypothalamic, amygdaloid and midbrain regions projecting to the VMH, and VMH^NMU^ neurons projecting to anterior and posterior areas, from the LS and ST to the PAG and EW. The connectional architecture of the VMH has been extensively described using classical staining and tracing techniques [76–78]. The pattern described is consistent with previous literature, describing major afferents from the mammillary peduncle, amygdaloid body and LH [77,78] and a remarkable intrahypothalamic connectivity, from and to the anterior hypothalamus (mainly AHA and MPA), LH and PH [76–78]. The connectivity reported here partially overlaps with the connectional architecture reported for vlVMH^Esr1^ neurons [79], including projections to the BNST and hypothalamic (AHA, MPA, DMH), thalamic (PVH) and midbrain (PAG) regions. Interestingly, we found a substantial amount of NMU neurons in the vlVMH overlapping with ER*α*, supporting the hypothesis that NMU plays a modulatory role in reproduction and energy expenditure [2,80,81]. However, NMU neurons were found not only in the vlVMH, but throughout all three subdivisions of the VMH, in contrast with many other neuronal sub-populations identified in this nucleus, such as the aforementioned ER*α*, leptin receptor, pituitary adenylate cyclase-activating peptide (PACAP) or cholecystokinin receptor B (CCKBR), among others [82,83]. The expression pattern of NMU neurons in the VMH and ARC, the limited colocalization with ER*α* in both regions and the absence of colocalization with other well-described hypothalamic markers, such as POMC and NPY, suggest that NMU neurons constitute mainly a unique population of hypothalamic cells. Overall, the expression of NMU in the VMH and the connectional pattern to and from VMH^NMU^ support the idea of a midline NMU circuit with the VMH as a key node. In that sense, there is a relevant overlap between neuroendocrine functions attributed to the VMH and those described for NMU. The VMH is considered an integral region in many neuroendocrine functions, such as metabolic regulation and energy homeostasis, thermoregulation, bone remodelling as well as stress and anxiety-related behaviours [84,85], functions that have also been described for NMU [4]. A central stress-integrative system involving forebrain, amygdaloid, hypothalamic and midbrain region has been reported [86], pointing out a role of the VMH and PH outputs to the PVH in the regulation of the stress response. The suggested NMU midbrain circuit comprises most of those regions; certainly, a role of NMU in the modulation of the stress response in rodents has been extensively addressed [29,87–93]. It may be interesting to perform a detailed analysis of the connectivity between these NMU-expressing regions to gain insights into the connectional architecture of this potential NMU circuit. In addition, the presence of RFP-marked cells in hypothalamic and midbrain regions constitutes an additional validation of the mouse model, since these results confirmed Cre activity in adult life in regions where the reporter ZsGreen1 protein and *Nmu* mRNA were detected. Furthermore, the specific targeting of NMU neurons with the double-floxed viral tracer opens the door to the use of chemogenetic and optogenetic interventions in Nmu-Cre mice to modulate specific sub-populations of NMU-expressing neurons. This may allow further exploration of the role of NMU in the mouse brain.

In contrast with classic neurotransmitters, neuropeptides were initially considered as transmitters of a modulatory nature [94]. Nowadays, it is well known that neuropeptides are signalling molecules widely distributed throughout the CNS where they commonly occur with, and are complementary to, classic neurotransmitters, controlling a wide range of behaviours [95]. However, the idea of obligate peptidergic neurons has also recently been addressed in the mouse brain [96]. Our results showing co-expression of transcripts for fast-acting neurotransmitters and *Nmu* mRNA in VMH (NMU) neurons are consistent with the co-transmission described for many hypothalamic peptidergic neurons [95,97]. This co-expression was also found in another NMU-expressing region, the BNST. The effects of NMU are linked to two GPCRs, driving delayed changes in neuronal activity through a broad spectrum of signalling pathways. Although the functional effects of the co-transmission of neuropeptides and fast-acting neurotransmitters are still not fully understood [98], this phenomenon potentially gives NMU a much broader and diverse range of biological effects [95,99,100]. In addition, our data showed expression of the *Nmu* transcript following a punctate pattern not confined to perinuclear somatic regions, suggesting that local protein synthesis may play a role [101,102].

Taken together, our results strongly suggest that Cre expression in the Nmu-Cre knock-in mouse model largely and faithfully reflects NMU expression in the adult mouse brain, without altering endogenous *Nmu* expression. In addition, we provided a detailed whole-brain characterization of NMU-expressing neurons. Our results revealed a potential midline NMU modulatory circuit with the VMH as a key node. We believe that this mouse model can serve as a useful tool for future investigations on NMU neurons in the CNS and the periphery.

## Data Availability

Image data are provided in the electronic supplementary material [103].
